# Feasibility and Safety Properties of Metabolic-Flow Anesthesia Driven by Automated Gas Control^®^ in Pediatric Patients: A Prospective Observational Study

**DOI:** 10.3390/medicina61050786

**Published:** 2025-04-24

**Authors:** Emre Sertaç Bingül, Meltem Savran Karadeniz, Emre Şentürk, İrem Vuran Yaz, Ayşe Gülşah Atasever, Mukadder Orhan Sungur

**Affiliations:** 1 Department of Anaesthesiology and Reanimation, Istanbul University Istanbul Faculty of Medicine, Millet St. Surgical Sciences Building, 34093 Istanbul, Turkey; 2 Department of Anaesthesiology, KU Leuven, 3000 Leuven, Belgium

**Keywords:** pediatric anesthesia, inhalation anesthesia, metabolic flow anesthesia, automated gas control, recovery, low-flow anesthesia

## Abstract

*Background and Objectives*: Metabolic-flow (<0.35 L/min) anesthesia is practiced more often as manufacturers provide newer technologies, yet the benefits of metabolic-flow anesthesia have not been fully investigated. This study aimed to investigate the feasibility and safety of automated gas control (AGC^®^) mode, which provides metabolic-flow anesthesia, in a pediatric population. *Materials and Methods*: Pediatric surgery patients between 1 and 10 years of age were included in this prospective observational trial. After intravenous induction and safe orotracheal intubation, AGC^®^ was initiated, and total sevoflurane consumption (mL) and wash-in speed-based sevoflurane consumption data were collected to measure feasibility. For safety, inspired (F_i_O_2_), alveolar (F_A_O_2_), and expired (F_E_O_2_) oxygen concentration data, and inspired and alveolar sevoflurane (F_i_Sevo and F_A_Sevo, respectively) concentration data, were recorded. Changes in fresh gas flow (FGF) throughout the procedure and postoperative recovery data were also compared. *Results*: A total of 130 patients were eligible for this study, and 121 patients were included in the analyses; 30 patients had a wash-in speed of 4 (WI4) and 91 patients had a wash-in speed of 8 (WI8) at follow-up. The total mean sevoflurane consumption was 9.35 ± 4.93 mL for a median surgery duration of 100 min. WI8 patients consumed more sevoflurane (9.92 ± 5.08 mL vs. 7.79 ± 4.19 mL, *p* = 0.04). At the 15th and 30th minutes, the FGF dropped under minimal flow and metabolic flow limits, respectively (*p* < 0.001). The times to extubation and obeying commands were shorter in WI8 patients (8 (5–10) vs. 11 (5–15) *p* = 0.03, and 9.5 (5–10.5) vs. 13 (9–17) *p* < 0.01). *Conclusions*: Maintenance with AGC^®^ may offer up to 40 h of anesthesia, considering that the volume of a sevoflurane bottle is 250 mL, reflecting exceptional savings compared to conventional anesthesia management. Metabolic flow anesthesia driven by AGC^®^ is feasible and safe in pediatric anesthesia practice.

## 1. Introduction

Low-flow anesthesia (LFA) is defined in the literature as the administration of anesthesia with a fresh gas flow (FGF) less than half of the patient’s minute ventilation, and in practical terms, FGF rates below 1 L/min are generally accepted as low-flow [1]. Semi-closed- or closed-circuit techniques are considered to cause rebreathing to some degree, which has led clinicians to use higher flows as a common practice. However, with modern anesthesia machines, controversy regarding the safety of such anesthetic approaches has been ameliorated after many studies, and LFA has been incorporated into routine anesthesiology practice due to its additional advantages, such as decreased volatile agent consumption and greenhouse gas burden [2]. Without a doubt, the effect of cost-saving via volatile consumption reduction is greatest within the pediatric age group, since higher volatile concentrations are required to reach the desired MAC; additionally, LFA is suitable for use in large tertiary centers [3]. A lower FGF may be more applicable for adult patients because as the flow rate drops, the provision of adequate fractional oxygen in the gas mixture may be compromised, which makes LFA questionable for pediatric anesthesia.

A newer term, “metabolic flow”, which indicates an FGF lower than 0.35 L/min, has been gaining popularity [1]. The most advanced anesthesia machines are being advertised for their automatic gas flow adjustment properties, one of which is automated gas control^®^ (AGC^®^), which has the ability to lower the FGF to metabolic flow limits while adjusting the gas flow automatically [4]. AGC^®^ enables setting the end-expiratory volatile agent concentration or minimum alveolar concentration (MAC) to a target value. This overcomes the need to frequently adjust the vaporizer, volatile concentration, and gas flow. It has been shown that under low-flow conditions, anesthetists may struggle to provide an adequate MAC when they make the adjustments themselves [5]. Machines with AGC^®^ allow physicians to use this automatic feature by selecting from eight wash-in speeds (1—the slowest; 8—the fastest). Although this proposed feature appears to be favorable, perioperative clinical investigations are needed to determine if it is more cost-effective than LFA, and equally safe.

This study aimed to investigate the feasibility and safety of metabolic flow anesthesia driven by AGC^®^ in pediatric patients by observing changes in total volatile agent consumption and the inspired oxygen fraction (F_i_O_2_) throughout surgery. The effects of different wash-in speeds (with an initial high flow to saturate the whole circulatory system and provide steady-state inhalational anesthesia) on safety and recovery were also investigated. The primary outcome was the total amount of sevoflurane consumed. The secondary outcomes included oxygen concentration parameters, which refer to set (F_i_O_2_), alveolar (F_A_O_2_), and expired (F_e_O_2_) values, and the times to extubation and cooperation after stopping volatile agent inhalation.

## 2. Materials and Methods

### 2.1. Study Population and Regulatory Aspects

**Figure 1:**
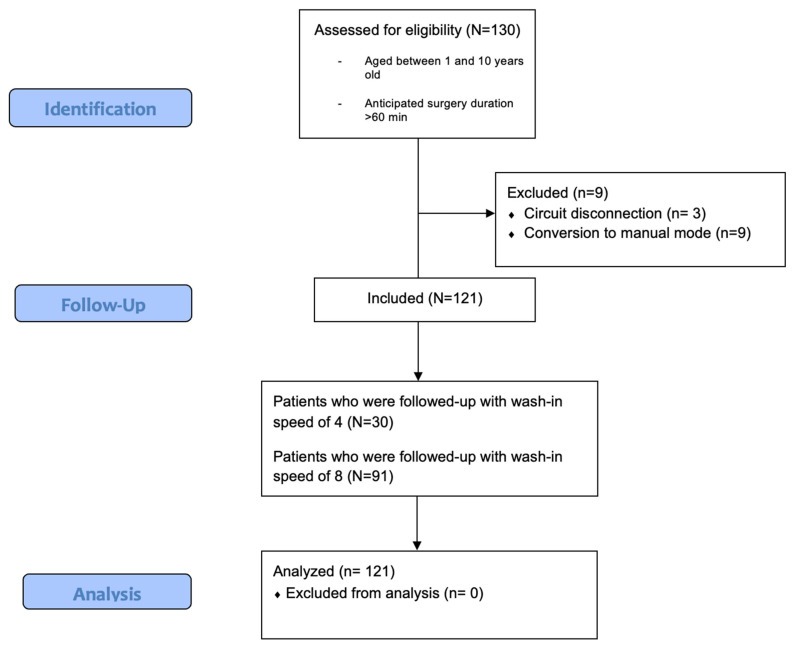
Flow diagram of the study.

This prospective, single-center, observational study was approved by the local ethics committee (Istanbul University Istanbul Faculty of Medicine Clinical Research Ethics Committee-2019/154) and registered at ClinicalTrials.gov (NCT:05644340). The current design is compatible with STROBE guidelines (Figure 1). The participants were children between 1 and 10 years of age with American Society of Anesthesiology Physical Status I-II who presented at the pediatric surgery department of our tertiary hospital and underwent surgery under general anesthesia with an anticipated duration of more than 60 min between December 2022 and April 2023. Informed consent was obtained from all patients’ legal guardians prior to their enrollment. Patients with known myopathy, risk of malignant hyperthermia, and who were contraindicated for inhalational anesthesia were excluded from this study.

### 2.2. Anesthetic Management

All patients received standard premedication with oral midazolam 0.5 mg/kg in the preoperative admission room, where an intravenous (IV) cannula was inserted right after applying EMLA 5% (Sanofi, Kırklareli, Turkey). After their arrival in the operating room, the patients were monitorized according to standard ASA recommendations to observe electrocardiography, pulse oximetry, end-tidal carbon dioxide, temperature, and blood pressure noninvasively. General anesthesia was induced intravenously with fentanyl 2 mcg/kg IV (Haver Pharma, Istanbul, Turkey), propofol 2% 2–3 mg/kg IV (Polifarma, Tekirdağ, Turkey) and rocuronium 0.5 mg/kg IV (Vem, Tekirdağ, Turkey) via the IV cannula. After adequate hypnosis and muscle relaxation, patients were intubated orotracheally, and mechanical ventilation (FLOW-i^®^, Maquet, Solna, Sweden) was initiated. No volatile agent was used in anesthesia induction.

After safe intubation, AGC^®^ was activated, targeting an MAC of 1.2. As the AGC^®^ software (Ver. 4.0.0) is capable of determining the gas flow, expiratory sevoflurane concentration, and F_i_O_2_ independently, the researchers did not adjust any other parameter during the surgery. The selection of wash-in flow speeds was left to the attending anesthetists’ (MSK, MOS) preference and they were evaluated after the completion of the study. Ventilatory settings maintained end-tidal CO_2_ (EtCO_2_) within normal limits using the volume-controlled mode (PEEP, 4 cmH_2_O; tidal volume, 6–8 mL/kg; frequency, 14–24 per minute; EtCO_2_, 30–40 cmH_2_O). A threshold of a 35% expired oxygen fraction (F_e_O_2_) was defined to be safe for this trial, and an F_e_O_2_ lower than 35% was considered to indicate hypoxic gas mixture delivery by the system. As a routine procedure, paracetamol 15 mg/kg IV was administered at the time of surgical skin closure for postoperative pain management. Once the surgery was completed, the expiratory sevoflurane concentration was set to zero, which would determine the pace of recovery according to the selected wash-in speed of automated gas flow. To reverse the neuromuscular blockade, sugammadex 2 mg/kg IV was administered to all patients, and extubation was performed after clinical findings (in patients capable of maintaining SpO_2_ above 95% with the presence of eye opening, conjugate gaze, facial grimacing, or purposeful movement) met the necessary criteria [6].

### 2.3. Outcome Measurement

The data were collected by members of the research team (ESB, EŞ, and İVY). The primary outcome, feasibility, was measured based on the total sevoflurane consumption, which was recorded at the end of the surgery and evaluated in the whole patient group, and by comparing wash-in speeds. Safety properties were demonstrated based on FGF, F_A_O_2_, F_i_O_2_, and F_e_O_2_ data, which were evaluated at certain time points (at 15, 30, 45, 60, 75, and 90 min after intubation). The time to reach the targeted expiratory volatile concentration (F_e_Sevo), time to reach low-flow threshold (minutes), and time to reach metabolic flow threshold (minutes) were recorded. Fractions of sevoflurane, which were both set (F_i_Sevo) and measured (F_A_Sevo) by the system, were recorded. To demonstrate recovery from inhalation anesthesia, the lengths of time after setting the targeted MAC to zero (end of surgery) until “extubation” and “obeying commands” were obtained. Patients older than 3 years of age were analyzed, since children in this age group are able to cooperate in the evaluation of “time to obeying commands” data.

### 2.4. Statistical Analysis

Based on Carette et al.’s study [4], assuming 8 ± 1 mL of sevoflurane consumption for an average 60 min surgery and possible 5% change in the consumption, the study design included 100 patients for 80% power and an alpha value of 0.05. Considering 20% drop-out, at least 120 patients were required.

Categorical data were expressed as numbers and percentages, which were compared with the chi-square test. Skewness and kurtosis were used to determine the distribution of quantitative data. Normally distributed data were expressed as the mean ± standard deviation, which were analyzed using unpaired sample *t*-tests. Heterogeneously distributed data were expressed as the median (25th percentile–75th percentile) and compared using the Mann–Whitney U test. A two-way repeated ANOVA was used to examine the effect of time on normally distributed variables. For data that violated the sphericity assumption, Greenhouse–Geisser sphericity correction was used. The Friedman test was used to determine the effect of time on fresh gas flow data. Bonferroni correction was used for pairwise comparisons. Statistical significance was indicated by *p* < 0.05, and statistical analysis was performed using SPSS V21.0 (IBM, Chicago, IL, USA).

## 3. Results

In total, 130 pediatric patients were enrolled in this study. Three patients were excluded due to a circuit disconnection, which altered the gas equilibrium in the system, and 6 patients were excluded due to a conversion to manual mode during AGC^®^. Consequently, a total of 121 patients were included. Among these, follow-up was conducted on 30 patients for whom a wash-in flow speed of 4 (WI4) was used and 91 patients for whom a high wash-in flow speed of 8 (WI8) was used; these speeds were chosen by physicians and applied in AGC^®^ mode (Figure 1).

**Figure 2:**
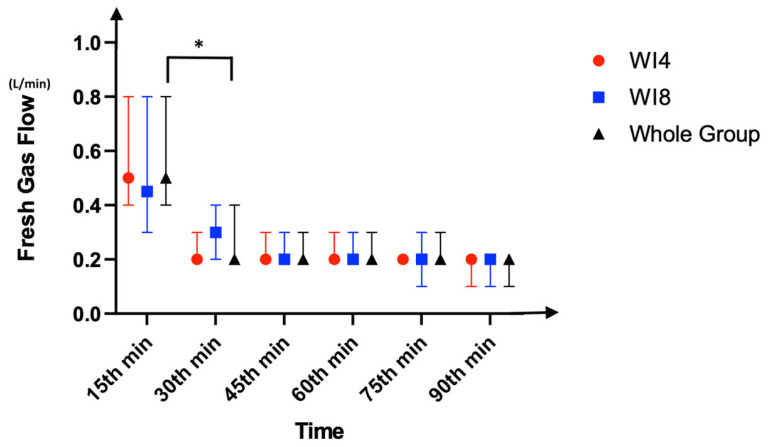
Timely fresh gas flow data for WI4, WI8, and whole group. Data are presented as median (IQR) values. *: *p* < 0.001.

**Figure 3:**
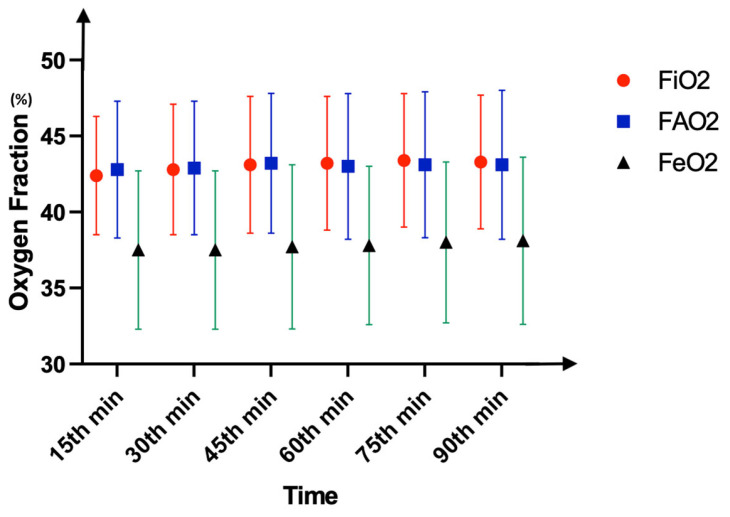
Inspired (F_i_O_2_), alveolar (F_A_O_2_), and expired (F_e_O_2_) fractions of oxygen in the whole group. Data are presented as median (IQR) values. Two-way repeated ANOVA did not exhibit any difference between parameters in both intergroup and intragroup analyses (*p* > 0.05).

**Figure 4:**
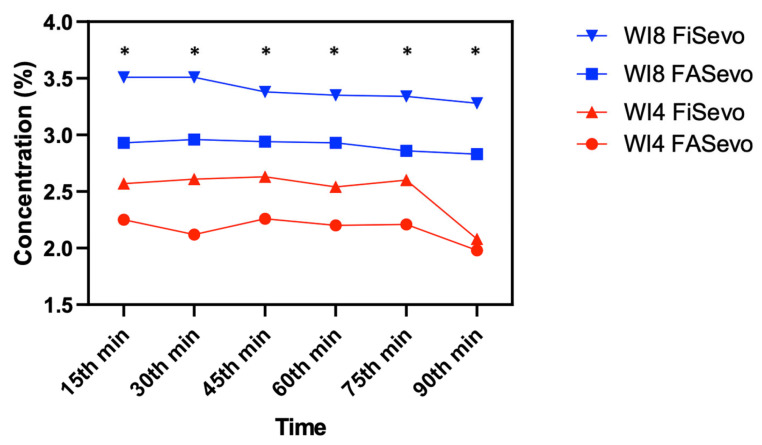
Alveolar (F_A_Sevo) and inspired (F_i_Sevo) concentrations of sevoflurane during surgery. Data are presented as median values. Sevoflurane concentrations were significantly lower in the WI4 group at every time point. *: *p* < 0.001.

Demographics are summarized in Table 1. The primary outcome, mean sevoflurane consumption in the whole group, was 9.35 ± 4.93 mL, and the total anesthesia durations were similar, with a median value of 100 min (*p* = 0.11). This indicates an approximate consumption of 6.2 mL per hour. In the group-based comparison, the mean sevoflurane consumptions were 7.79 ± 4.19 mL in WI4 and 9.92 ± 5.08 mL in WI8, with WI4 being significantly lower (*p* = 0.04). Pairwise analyses demonstrated significantly higher FGF levels at the 15th and 30th minutes when compared to later time points except for the time period between 30 and 45 min (*p* < 0.01). After the AGC^®^ mode was initiated, the fresh gas flow dropped below the minimal flow threshold (0.5 L/min) at the 15th minute and below the metabolic flow threshold (0.35 L/min) at the 30th minute (Figure 2). However, comparing the groups in terms of flow rate did not indicate any difference at those time points (15th minute, 0.5 L/min in WI4 vs. 0.45 L/min in WI8; 30th minute, 0.2 L/min in WI4 vs. 0.3 L/min in WI8, *p* > 0.05). As indicators of safety, the F_A_O_2_, F_i_O_2_, and F_e_O_2_ were similar between the groups and higher than 35% at all time points (*p* > 0.05) (Figure 3). The F_A_Sevo and F_i_Sevo levels were significantly lower in WI4 in comparison to WI8 at all time points (*p* < 0.001) (Figure 4). Moreover, the WI8 group patients demonstrated a faster recovery in terms of “time to extubation” (*p* = 0.03) and “time to obeying commands” (*p* < 0.001). Anesthesia-related data are presented in Table 2.

Hemodynamic parameters, including blood pressure and heart rate, did not exhibit any significant differences between the groups (*p* > 0.05). The data are presented in Table 3.

## 4. Discussion

In this single-center, prospective, observational trial, the automated gas control mode of the anesthesia machine provided cost-effective and safe anesthesia in children undergoing surgery. The expenditure of the volatile agent was even more remarkably low when the slower wash-in speed was chosen. Moreover, metabolic flow did not lead to the inspiration of hazardous alveolar oxygen fractions, reflecting a good safety profile, and hemodynamics were stable throughout the surgeries, regardless of the wash-in speeds.

The existing literature mostly focuses on medium- to high-flow anesthesia-related volatile expenditure, and conclusions are favorable regarding lower flows (1.5–3 L/min) [7,8,9,10]. However, with the exception of Carette et al.’s study, in which only an adult population was investigated, liquid volatile consumption analyses have not yet been performed for automated flows that have the ability to reach metabolic limits [4]. Carette et al.’s findings were slightly better than ours, possibly because of the limited patient number (*n* = 24), and their target was “F_A_ sevoflurane” (2.0%), not the MAC. Our results reflect an approximate sevoflurane consumption of 6.2 mL per hour when AGC^®^ mode is active in the whole-group analysis, indicating a possible 40 h of inhalation anesthesia per bottle (250 mL) in total. This number is quite remarkable when compared to Ryu et al.’s investigation. In this investigation, the authors calculated 13 h of inhalation anesthesia per 250 mL bottle after implementing a new institutional policy of low-flow anesthesia instead of the traditional approach, which produced almost 45% acquisition considering the previous total 9 h of lasting inhalational anesthesia per bottle [11]. Similarly, Singh et al. have almost halved sevoflurane consumption in pediatrics with LFA [12]. Notably, in both these studies, the adjustments were made manually by physicians targeting a flow of 1 L/min. At this point, we believe that automated flow may be a major determinant for volatile expenditure because it eliminates the need to frequently adjust both the vaporizer and FGF. Like our results, a comparative study by Tay et al. demonstrated a 27% cost reduction when the automated control of end-tidal gases was chosen over manual adjustment [13]. This reduction may be more prominent for surgeries shorter than 40 min, as Singaravelu and Barclay showed a major decrease from GBP 15 to GBP 7 per hour with an automated flow system in short surgeries [14]. In these abovementioned studies with automated systems, fresh gas flow varied between 0.5 L/min and 6 L/min, which is not below the metabolic threshold (0.35 L/min) [13,14]. Therefore, it is difficult to claim that metabolic flow is superior to low flow, and further research should focus on comparing these two entities. Nevertheless, a volatile consumption of around 6–7 mL per hour with AGC is remarkable and highly valuable for everyday practice. Notably, under AGC settings, the FGF occasionally drops to 0.1 L/min in longer surgeries, which would contribute even more to potential cost savings.

The wash-in speed is another determinant for reducing cost. Kalmar et al. have shown a 1 mL difference in volatile consumption between wash-in speeds of 4 and 8 during 45-min surgeries [15]. In the current study, about 1.5 mL of liquid per hour was saved when slower wash-in was chosen, and these data agree with the abovementioned study by Carette et al. [4]. De Medts et al. were able to demonstrate similar findings with desflurane [16]. Despite the sparsity of the literature, existing clinical data reliably indicate improvements in cost savings with a slower equilibration of the gas mixture in the system. This “slower” wash-in may not necessarily indicate a “disadvantage” for the surgical process; rather, it may actually create a time frame for pre-emptive regional blocks that could synergistically enhance the anesthesia, acting as another cost-saving entity. However, physicians in busy surgical environments may choose fast wash-in speeds, which still provide a great reduction in expenses. Notably, faster wash-in speeds did not translate into hemodynamic instability in our results.

LFA is mistakenly believed to cause a significant delay in emergence from inhalational anesthesia; however, clinical data derived by Stevanovic et al. show similar recovery times with low-flow volatile inhalation when compared to targeted, controlled propofol infusion [17]. In that study, adult laparoscopic cholecystectomy patients were compared, and the time to extubation and early emergence were significantly faster with LFA (6 vs. 8 min and 7.5 vs. 9 min, respectively). Pediatric patients would require relatively more volatile consumption because of the higher levels of concentrations needed to reach a targeted MAC, which would consequently increase the time for complete recovery. On the contrary, our results reveal recovery parameters that were within a time span (between 9 to 13 min) similar to that of Stevanovic et al.’s study. This may be attributed to the automated flow system because it can instantly change the volatile concentration to establish the desired MAC and end-expiratory volatile concentration. At this point, different wash-in speeds may be another matter of debate regarding the velocity of reaching inhalational steady state and duration to emergence. Faster wash-in resulted in the early stabilization of the alveolar concentration of the volatile agent, and emergence at the end of surgery occurred earlier. However, the possible clinical benefits remain uncertain since the time difference between the two wash-in speeds is only around 3 to 4 min.

**Figure 5:**
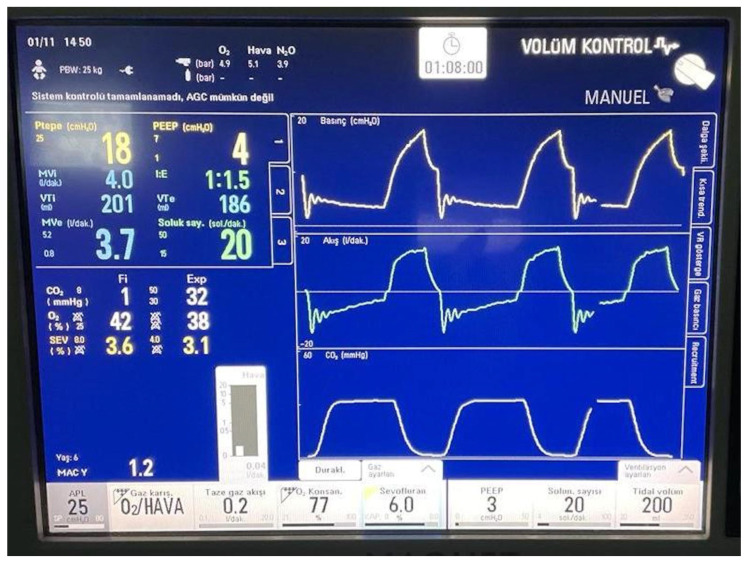
The interface of Maquet Flow-i in Automated Gas Control^®^ mode (in Turkish).

One of the major concerns with LFA is the drop in oxygen fraction, which may lead to unintentional hypoxic gas mixture inhalation. As the flow decreases, the F_A_O_2_ decreases to less than the adjusted amount [18,19]. Manufacturers appear to have overcome this issue by developing AGC^®^ software, which rapidly adjusts the F_A_O_2_. As seen in Figure 5, the F_A_O_2_ may reach quite high levels (77%) to provide safety, while the FGF was only 0.2 L/min. Therefore, we did not observe any undesirably deviated alveolar oxygen fractions during the surgeries at any time point. These data are also compatible with those from De Medts et al.’s study, in which the F_A_O_2_ did not drop below 30% when using an automated system in which the F_i_O_2_ was set according to a 35% standard [16].

We did not induce anesthesia via inhalation, which might be considered a limitation. However, doing so would change the perspectives of this study and the cost analysis, since the aim was to examine the economical outcomes of using automated gas systems in the first place. Induction via mask ventilation is associated with technical difficulties and a high possibility of circuit disconnection, which causes unintentional wastage and makes it difficult to calculate the costs objectively. However, in one study, Datta et al. were able to demonstrate a significant cost reduction for short procedures, such as ophthalmic examinations, in pediatrics when a minimal gas flow (0.5 L/min) after inhalational induction was chosen [20]. While this technique offers advantages in reducing costs, the application of low-flow anesthesia during induction remains impractical, especially in pediatric patients, with whom communication is challenging. Another limitation would be the lack of depth of anesthesia monitorization, which would enhance the reliability and correlation of our volatile monitoring and anesthetic depth findings. In the current study, the MAC value was traditionally standardized to 1.2 and adjusted according to the patient’s age. Although a randomized study would have been preferable, the primary objective was to observe the consistency between automated gas control and metabolic flow anesthesia. A randomized controlled trial should be considered as a potential next step for future research.

A paradigm shift towards the widespread usage of LFA in routine anesthesiology practice worldwide is developing [2,3,19,21]. The environment has become an utmost priority, driving physicians to protect it by reducing greenhouse gasses and pollution, and LFA seems to be one of the most valuable interventions for this purpose [1,22]. Automated end-tidal gas controlling systems deserve clinical attention, considering their eco-friendly, economical, and safe features.

## 5. Conclusions

Our experience with AGC^®^ in a pediatric population was that it was completely cost-effective, and the savings were even higher with slower wash-in speeds. As the literature suggests, metabolic-flow anesthesia is doubtless the most efficient set-up in both theoretical and clinical settings, and the automated flow systems in the latest technology are well-suited to such anesthetic implementation. Our study revealed a completely feasible and safe practice of driving metabolic flow via AGC^®^ in a pediatric surgery population.

## Figures and Tables

**Table 1:** Demographic and procedural data.

	Total (*N* = 121)		
Age (months)	60 (30–120)		
Weight (kg)	24.7 ± 16.1		
Female/Male (*n*(%))	34 (28)/87 (72)		
Surgery Type (*n*)			
Orchiopexy ± hernia repair	35		
Bilateral inguinal hernia repair	14		
Pyeloplasty	10		
Hypospadias repair	31		
Major oncologic surgeries	10		
Laparoscopic appendectomy	14		
Cyst hydatic extirpation	4		
Intussusception repair	3		
Surgery duration (min)	84 (81–101)		
	**WI4 (*N* = 30)**	**WI8 (*N* = 91)**	***p*** *
Age (months)	60 (30–110)	63 (28–120)	0.94
Weight (kg)	27.1 ± 15.4	23.8 ± 16.3	0.34
Female/Male (*n* (%))	12 (40)/18 (60)	22 (24)/69 (76)	0.18
Surgery Type (*n*)			0.14
Orchiopexy ± hernia repair	7	28	
Bilateral inguinal hernia repair	3	11	
Pyeloplasty	2	8	
Hypospadias repair	10	21	
Major oncologic surgeries	2	8	
Laparoscopic appendectomy	4	10	
Cyst hydatic extirpation	1	3	
Intussusception repair	1	2	
Surgery duration (min)	82 (81–85)	84 (81–110)	0.13

Data are expressed as numbers (*n*), percentages (%), means ± standard deviations, medians (interquartile range), and minutes (min). *: Statistical analyses were between-group comparisons.

**Table 2:** Anesthesia-related data.

	WI4 (*N* = 30)	WI8 (*N* = 91)	Total (*N* = 121)	*p* *
Duration of anesthesia (min)	99 (96–103)	101 (95–122)	100 (95–113)	0.11
Target F_e_Sevo (%)	2.20 ± 0.30	2.53 ± 0.51	2.30 ± 0.39	0.06
Time to reach targeted F_e_Sevo (min)	10 (8–12)	2 (2–2)	2 (2–4)	<0.001
Total sevoflurane consumption (mL)	7.79 ± 4.19	9.92 ± 5.08	9.35 ± 4.93	0.04
Volatile closure to extubation (min)	11 (5–15)	8 (5–10)	10 (5–12)	0.03
	**WI4 (*n* = 16)**	**WI8 (*N* = 39)**	**Total (*N* = 55)**	
Volatile closure to obey commands (min)	13 (9–17)	9.5 (5–10.5)	10 (5–14)	<0.01

Data are expressed as percentages (%), milliliters (mL), means ± standard deviations, medians (interquartile range), and minutes (min). *: Statistical analyses were provided as between-group comparisons.

**Table 3:** Median values of hemodynamic parameters.

Heart Rate (beat/min)	Induction	15 min	30 min	45 min	60 min	75 min	90 min	End of Surgery
WI4	121.5 (110–130)	115 (100–128)	109 (91–127.5)	99.5 (90–113)	98.5 (87–116)	93 (77–106)	92.5 (78–105)	93 (90–108)
WI8	112 (106–131)	102 (99–121)	104 (98–118)	101 (93–109)	100.5 (95–110)	102 (99–109)	100 (98–108)	101 (91–108)
*p* *	0.7	0.4	0.9	0.7	0.8	0.1	0.2	0.8
**Mean Arterial Pressure (mmHg)**								
WI4	78 (74–84)	71 (65–75)	65 (62.5–69)	66 (62–70)	67 (64–69)	64.5 (62–68.5)	61 (62–67)	61 (59–62)
WI8	77 (70–82)	70 (66–79)	70 (63–73.5)	68 (62.5–70)	63.5 (61–66.5)	64.5 (60–67)	63 (62–65)	61 (60–64)
*p* *	0.5	0.7	0.2	0.5	0.1	0.6	0.4	0.7

Data are presented as median (interquartile range) values. WI4: Wash-in speed 4 group. WI8: Wash-in speed 8 group. * Mann–Whitney U test.

## Data Availability

The clinical data from this trial are available from the corresponding author upon reasonable request.

## Abbreviations

AGC^®^	Automated gas control
EtCO_2_	End-tidal carbon dioxide
F_A_O_2_	Alveolar fraction of oxygen
F_E_O_2_	Expiratory fraction of oxygen
F_i_O_2_	Inspiratory fraction of oxygen
F_A_Sevo	Alveolar fraction of sevoflurane
F_i_Sevo	Inspiratory fraction of sevoflurane
FGF	Fresh gas flow
IV	Intravenous
LFA	Low-flow anesthesia
MAC	Minimum alveolar concentration
PEEP	Positive end-expiratory pressure
STROBE	Strengthening the Reporting of Observational Studies in Epidemiology
WI4	Wash-in speed of four
WI8	Wash-in speed of eight
